# Liver Metabolomics Reveals the Effect of *Lactobacillus reuteri* on Alcoholic Liver Disease

**DOI:** 10.3389/fphys.2020.595382

**Published:** 2020-11-12

**Authors:** Tian-xiang Zheng, Shi-lin Pu, Peng Tan, Yi-chao Du, Bao-lin Qian, Hao Chen, Wen-guang Fu, Mei-zhou Huang

**Affiliations:** ^1^Department of Hepatobiliary Surgery, The Affiliated Hospital of Southwest Medical University, Luzhou, China; ^2^Academician (Expert) Workstation of Sichuan Province, The Affiliated Hospital of Southwest Medical University, Luzhou, China

**Keywords:** alcoholic liver disease, alcoholic hepatitis, *Lactobacillus reuteri*, metabolomics analysis, fatty acid metabolism

## Abstract

Alcoholic liver disease (ALD), a type of chronic liver disease that is prevalent worldwide, is still identified to have a poor prognosis despite many medical treatment protocols. Thus, it is urgent to develop and test new treatment protocols for ALD. *Lactobacillus reuteri* (*L. reuteri*) has been widely used in the clinical treatment of digestive system diseases, but studies on the protective effect of *L. reuteri* on ALD are considered to be rare. Therefore, in the present study, we examined the effect of *L. reuteri* on ALD and provide data that are significant in the development of new treatment protocols for ALD. An ALD model has been established in C57BL/6J mice treated according to the Gao-binge modeling method. Mice in the treatment group were administered with *L. reuteri*. Hematoxylin and eosin (H&E) staining, oil red O staining, immunohistochemistry, and biochemical analyses were performed to detect the phenotypic changes in the liver among mice in the different treatment groups. *L. reuteri* treatment reversed inflammatory cell infiltration and lipid accumulation. Moreover, AST, ALT, TG, and TCH levels were also reduced in the probiotics-treatment group. Five candidate biomarkers were found in the liver metabolites of different treatment groups by UPLC/QTOF-MS and a multivariate analysis. Several fatty acid metabolic pathways such as linoleic acid metabolism and glycerolipid metabolism were involved. All these findings suggested that *L. reuteri* treatment reversed the phenotype of ethanol-induced hepatitis and metabolic disorders. These findings provide evidence that *L. reuteri* might serve as a new therapeutic strategy for ALD.

## Introduction

Alcoholic liver disease (ALD) has been identified as one of the most common liver diseases worldwide. According to the latest World Health Organization estimates, 3,000,000 alcohol-related deaths, were reported in 2016, which accounted for 5.1% of the global disease burden (World Health Organization, 2018). Alcohol can reportedly inhibit mitochondrial β-oxidation, increase fatty acid synthesis, and accumulate fatty acids in the liver, which results in the perturbation of lipid metabolism homeostasis. ALD is also underlying cause of many liver diseases, including alcoholic steatohepatitis (ASH) and hepatocellular carcinoma (HCC). ASH is an advanced stage of ALD, characterized by chronic liver injury, inflammation, and fibrosis and can ultimately progress to HCC (Seitz et al., 2018). Corticosteroids and pentoxifylline (TNF-α inhibitor) are commonly used to treat ASH, but they fail to decrease the risk of 6-month mortality (Singh et al., 2015; Thursz et al., 2015). Therefore, the discovery of a new feasible approach for the treatment of ALD is urgently needed.

In recent years, the gut microbiome has attracted increased attention in the field of metabolic diseases. *Lactobacillus reuteri*, a type of probiotic, has been demonstrated to be beneficial in many diseases, including diarrhea and infant colic (Jimenez-Gutierrez et al., 2014; Chau et al., 2015). *L. reuteri* has been reported to inhibit Gram-negative bacteria by secreting reuterin, a well-known antimicrobial compound, and several other antimicrobial substances, such as reutericyclin, lactic acid, and acetic acid (Yang et al., 2015; Greifova et al., 2017). It has been demonstrated that *L. reuteri* can attenuate multi-organ inflammation while remodeling the gut microbiome of *foxp3*-mutant mice with gut microbial dysbiosis (He et al., 2017). Many studies on obesity, type 2 diabetes, and fatty liver disease have shown that *L. reuteri* treatment reversed insulin resistance, hepatic steatosis, and inflammation (Hsieh et al., 2013; Marie-Christine et al., 2015; Dahiya et al., 2017).

Alcoholic liver disease is also a type of metabolic disease. The commonly used methods of histological analysis and the detection of several biochemical biomarkers lack the comprehensive recognition of metabolic changes. Metabolomics analysis is an indispensable and readily used method that can detect changes in metabolism in response to various stimuli by analyzing alterations in endogenous small molecule metabolites (Noorbakhsh et al., 2019). Therefore, metabolomics analysis is determined to be superior in the detection of liver metabolites, guiding clinical diagnosis and determination of prognosis. Few studies have focused on the metabolic effect of *L. reuteri* on ALD. Thus, a metabolomics analysis was performed in this study. We also hypothesize that *L. reuteri* treatment can reverse the progression of ALD by ameliorating disorders of liver metabolism.

## Materials and Methods

### Animals

Male C57BL/6 mice (8–10 weeks of age) were obtained from Chengdu Dashuo Biotechnological Company. A Lieber-DeCarli liquid ethanol diet and a control diet were purchased from TROPHIC Animal Feed High-tech Co., Ltd., China. Edible alcohol (95%) was purchased from Henan Xinheyang Alcohol Co., Ltd. *L. reuteri* DSM17938 was purchased from BioGaia. All mice were housed in a specific pathogen-free environment (temperature 23°C ± 2°C, humidity 55% ± 5%, and a 12-h light/dark cycle) with free access to water and the liquid ethanol diet or the control diet.

Eighteen male mice were randomly assigned to three groups. The control group was fed a control diet (TROPHIC Animal Feed High-tech Co., Ltd., Nantong, China); meanwhile, the model and probiotics-treatment groups were fed an ethanol diet (TROPHIC Animal Feed High-tech Co., Ltd., Nantong, China). Moreover, the model and probiotics-treatment groups were administered edible alcohol by oral gavage (0.25 g/ml ethanol, 5 g/kg of body weight) weekly. In addition, probiotics-treatment group was, respectively, treated with *L. reuteri* (2 × 10^7^ CFU, daily). After 8 weeks, the mice were sacrificed by isoflurane inhalation (3–4%). Another group of five mice was treated by control diet and *L. reuteri* (2 × 10^7^ CFU, daily) for 8 weeks to access the safety of *L. reuteri*. Different groups were given equal amounts of ethanol diet precisely daily, and the residual was recorded the next day and the amount of food was adjusted to make sure they ingested a similar amount of alcohol. All experiments were approved by the Animal Care and Use Committee of Southwest Medical University (approval No. XNYKDX202005) and were performed in accordance with the Guidelines for the Care and Use of Laboratory Animals issued by the United States National Institutes of Health.

### Sample Collection and Preparation

Plasma samples were collected and centrifuged at 10,000 rpm for 10 min at 4°C, and the resultant supernatants were stored at −80°C for biochemical analysis. Liver and intestinal tissues were removed immediately and later placed in phosphate-buffered saline solution for cleaning. Then, the samples were stored at −80°C for histologic analyses. To perform the metabolomics analysis, 0.2 g of each liver sample was added to 2 ml of a methanol and water (4:1) mixture, homogenized for 1 min, extracted by ultrasonication on an ice water bath for 8 min (80 Hz), and filtered through a 0.22-μm nylon filter in order to obtain 1.6 ml of supernatant.

### Detection of Biochemical Biomarkers in Serum

The levels of serum aspartate aminotransferase (AST), alanine aminotransferase (ALT), alkaline phosphatase (ALP), triglycerides (TG), and total cholesterol (TCH) were detected using an automatic biochemical analyzer (Erba XL-640, Germany).

### ELISA of Lipopolysaccharide (LPS) and TNF-α in Serum

The ELISA kits of Lipopolysaccharide (LPS) and TNF-α in serum were purchased from Bioswamp (Wuhan, China).

### Liver Morphology and Intestinal Tight Junctions

Liver and intestinal samples were fixed in 4% paraformaldehyde for 24 h, embedded in paraffin, and cut into sections. The sections were examined by light microscopy (100 ×, 400 ×) after hematoxylin and eosin (H&E) staining, oil red O staining, and immunohistochemical staining for ZO-1 and occludin according to the manufacturer’s protocols.

### Metabolomics Analysis

#### Chromatographic Conditions

The liver metabolomics analysis was performed using an Agilent 1290 ultrahigh-pressure liquid chromatography machine (Agilent Technologies, Palo Alto, CA, United States). ZORBAX Eclipse Plus C18 RRHD columns (2.1 × 150 mm, 1.8 μm) with a temperature that was maintained at 35°C were used in this analysis. The mobile phase was composed of A (0.1% formic acid in water) and B (0.1% formic acid in acetonitrile). The flow rate of the mobile phase was set at 0.4 ml/min. The linear gradient elution program was set as follows: at 0–2 min, 98% A and 2% B; 2–9 min, 98% A and 2% B; 9–15 min, 55% A and 45% B; 15–22 min, 30% A and 70% B; 22–24 min, 2% A and 98% B; and 24–26 min, 98% A and 2% B.

#### Mass Spectrometry Conditions

The metabolomics analysis was performed using an Agilent 6530 quadrupole time-of-flight Mass Spectrometer (Agilent Technologies, Palo Alto, CA, United States), which was equipped with an electrospray ionization (ESI) source. The positive and negative modes were used to collect the data from 50 to 1000 m/z at a rate of 1 spectra/s. The optimal conditions were set as follows: fragmentation voltage: 135 V; the skimmer voltage: 65 V; capillary voltage: ESI + : 4.0 kv, ESI-: 3.5 kv; desolvation gas flow: nitrogen, 10 L/min, 350°C, atomizer pressure: 45 psig.

#### Multivariate Statistical Analysis of the Serum Metabolite Data

MassHunter Qualitative Analysis (Agilent Technologies, United States) was applied to transform the original mass spectrometry data into the general mzData format, which was obtained from the LC-MS analysis. In order to obtain the generated data matrix, which consisted of m = z, retention time, and peak area, XCMS was employed for data processing such as peak recognition, filtering, and alignment. The generated data matrix was then imported to SIMCA (version 13.0, Umetrics AB, Sweden) to perform the multivariate statistical analysis by principal component analysis (PCA) and orthogonal partial least squares discriminant analysis (OPLS-DA); then, the result was obtained in the form of a score spot. The R^2^X, R^2^Y, and Q^2^ parameters were introduced to describe the result of OPLS-DA to obtain Variable Importance in Projection (VIP) using the permutation test in case of model overfitting and a *t*-test in order to evaluate statistical significance. The biomarkers were further screened in accordance with a VIP value >1, *p* < 0.05, and fold changes >1.5 or < 0.5.

#### Biomarker Identification and Metabolic Pathway Analysis

Human Metabolome Database (HMDB) was used to infer the possible molecular formula of compounds according to m = z and the relative isotopic abundance of the molecular ion peak. The molecular ion peaks were analyzed by secondary mass spectrometry under a different collision energy to obtain accurate fragment information. The final compounds were determined according to their accurate molecular ion mass, fragment information, and isotope abundance by interpreting the data according to databases such as the Human Metabolome Database (HMDB) and the Kyoto Encyclopedia of Genes and Genomes (KEGG).

### Statistical Analysis

Statistical analysis was performed using SPSS 17.0. *T*-test was used in the OPLS-DA model to ensure the reliability of the results. Differences of the body weight, liver weight, liver/body weight ratio, biochemical biomarkers, LPS and TNF-α in serum among three groups were compared using one-way analysis of variance with Tukey’s test. Data are expressed as the mean ± SD. All differences were considered statistically significant when *p* < 0.05.

## Results

### Establishment of the Mouse Model

**FIGURE 1:**
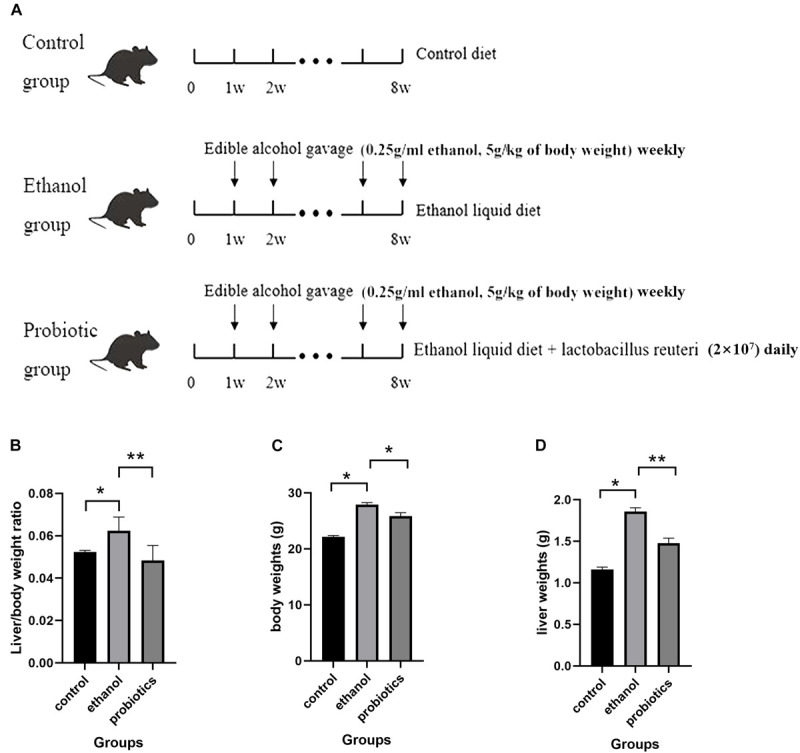
**(A)** Diagram of the mouse model. Mice were divided into three groups: control group, the ethanol group, and the probiotics group. The control group was fed a control diet. The ethanol group and probiotics group were fed a liquid ethanol diet and were administered edible alcohol via oral gavage (0.25 g/ml ethanol, 5 g/kg of body weight) weekly. Moreover, mice in the probiotics group were also treated with *L. reuteri* (2 × 107 CFU, daily). **(B)** Liver to body weight ratio of the three groups. **(C)** Body weight of three groups. **(D)** Liver weight of three groups. *p < 0.05, **p < 0.01.

To determine whether *L. reuteri* exerts protective effects on ALD, 18 male C57BL/6 mice were divided into 3 groups (Figure 1A). The control group was fed a control diet, while the ethanol group and the probiotics-treatment group were both fed a liquid ethanol diet and were administered edible alcohol via oral gavage (0.25 g/ml ethanol, 5 g/kg of body weight) weekly. *L. reuteri* was added to the drinking water in the probiotics-treatment group (2 × 10^7^ CFU, daily). Compared with the control group, mice, which were fed an ethanol diet, showed a significant increase in liver/body weight ratio, body weight and liver weight, while these increases were significantly reversed by *L. reuteri* treatment (Figures 1B–D).

### *L. reuteri* Treatment Ameliorated the Steatohepatitis Phenotype and the Expression of Intestinal ZO-1

**FIGURE 2:**
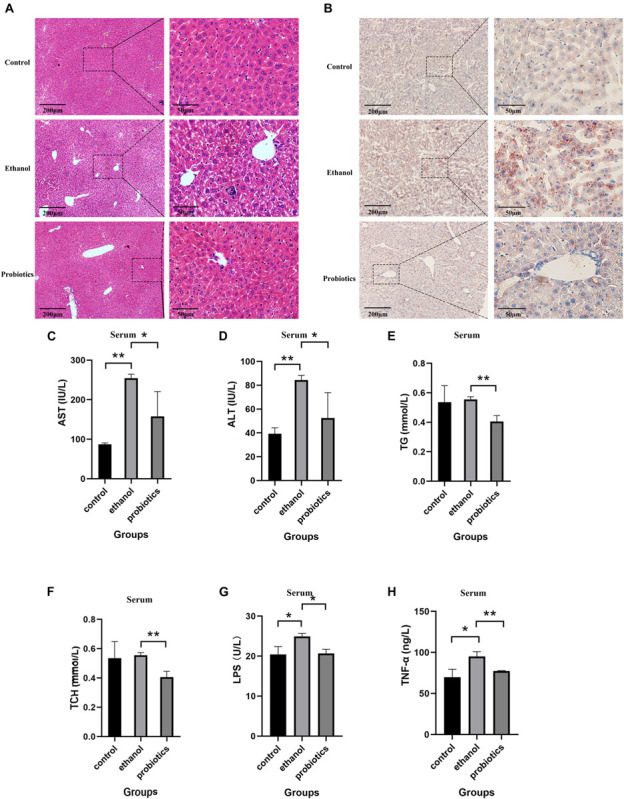
*L. reuteri* treatment reduces the ethanol-induced inflammatory cell infiltration, hepatic lipid accumulation, and liver injury. **(A,B)** H&E staining and oil red O staining in the three groups (100 × and 400 × magnification). **(C–F)** Biochemical analysis of serum samples. **(G,H)** Serum LPS and TNF-α ELISA. AST: aspartate aminotransferase, ALT: alanine aminotransferase, TG: triglycerides and TCH: total cholesterol. *p < 0.05, **p < 0.01.

To further detect the effects of *L. reuteri* on ALD, H&E staining and oil red O staining were performed to detect the histomorphological changes of steatohepatitis induced by an ethanol diet. Inflammatory cell infiltration and lipid vacuolation were observed in the livers of mice fed an ethanol diet (Figure 2A), and the levels were significantly determined to be different from those in mice in the control group. Oil red O staining also revealed accumulation of lipid droplets (Figure 2B). We then performed a serum biochemical analysis to explore the effects of *L. reuteri*. Compared with mice fed an ethanol diet, probiotics-treated mice showed an amelioration of liver injury, which manifested as a decrease in AST and ALT levels (Figures 2C,D). In addition, the biochemical analysis showed an accumulation of TG and TCH, which was consistent with the histologic analyses (Figures 2E,F). Serum LPS and TNF-α ELISA were performed to further detect the protective effect of *L. reuteri* treatment on liver (Figures 2G,H). To confirm the safety of *L. reuteri*, a group of mice was treated by control diet and *L. reuteri* in drinking water. The body weight, liver weight, liver/body weight ratio, HE and oil red o staining and other biochemical biomarkers in serum were compared with control group and the results showed no significant difference between these two groups (Supplementary Materials). The results indicated that *L. reuteri* treatment reversed the endotoxemia and inflammation induced by ethanol. These findings clearly indicated that the phenotype of steatohepatitis induced by ethanol was ameliorated using *L. reuteri* treatment.

**FIGURE 3:**
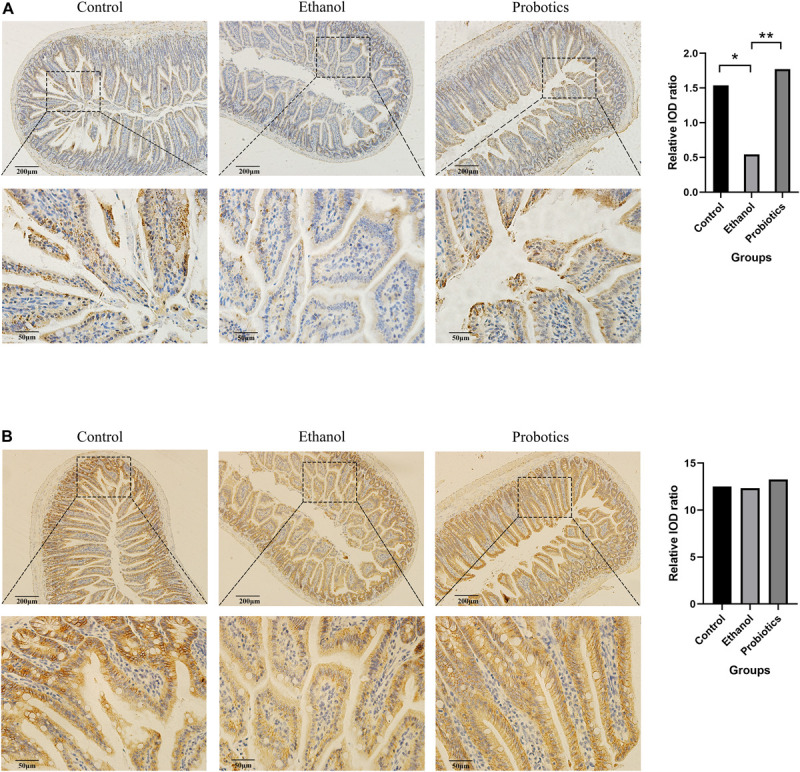
Immunohistochemical staining of intestinal tight junctions. **(A)** ZO-1 expression was significantly repressed in mice fed an ethanol diet and was reversed by *L. reuteri* treatment. **(B)** Occludin expression was not significantly different among the three groups (100 × and 400 × magnification). *p < 0.05, **p < 0.01.

*Lactobacillus reuteri* has been reported to be beneficial for the expression of intestinal tight junction (TJ) proteins in colitis, which improves gut barrier function and might affect ethanol-induced hepatitis (Ahl et al., 2016). Therefore, TJ protein expression was detected in the three groups using immunohistochemical staining. ZO-1 expression was significantly suppressed in mice fed an ethanol diet, which was reversed by *L. reuteri* treatment, while occludin expression was not significantly different among the three groups (Figures 3A,B).

### *L. reuteri* Reversed Polyunsaturated Fatty Acid Metabolism Disorder

**FIGURE 4:**
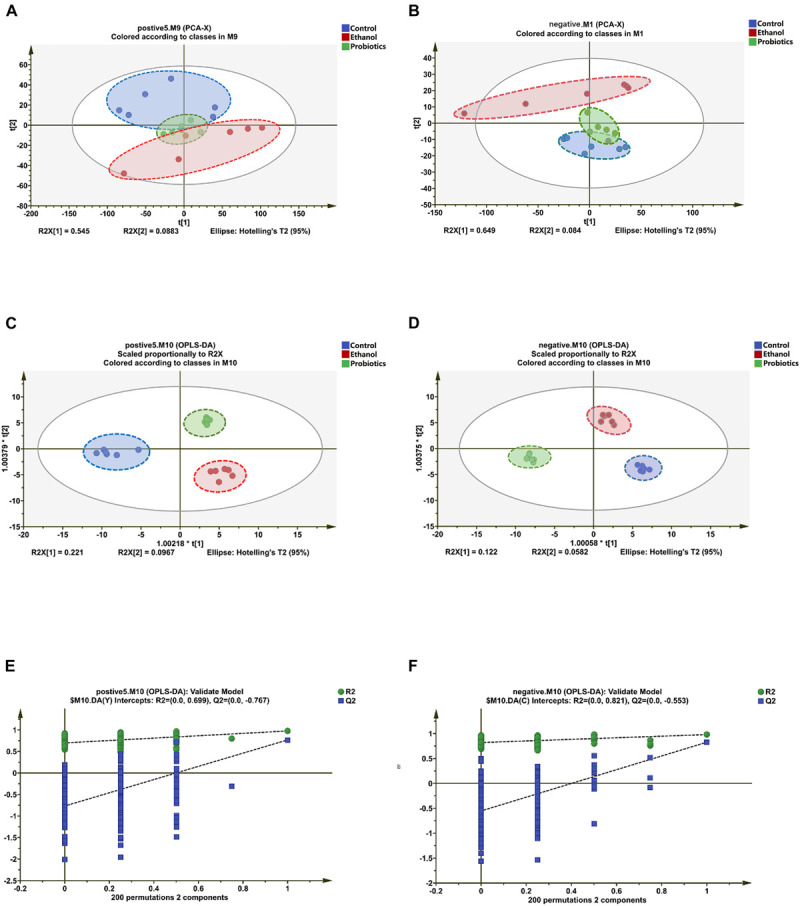
Differentiation of the liver metabolic profiles of the control group, ethanol group, and the probiotics group. **(A,B)** PCA score plots based on the liver metabolic profiles of the three groups in the positive and negative modes: ESI + : R2 = 0.545, ESI-: R2 = 0.649. **(C,D)** OPLS-DA score plots of the three groups in the positive and negative modes: ESI + : R2X = 0.735, R2Y = 0.995, Q2 = 0.728; ESI-: R2X = 0.711, R2Y = 0.974, Q2 = 0.792. **(E,F)** Permutation test of the OPLS-DA model: ESI + : the intercepts of R2 = 0.699 and Q2 = -0.767, ESI-: the intercepts of R2 = 0.821 and Q2 = −0.553.

To further explore the way in which *L. reuteri* reversed the progression of ALD, especially the metabolic changes, we performed a metabolomics analysis of mouse liver samples. UPLC Q-TOF/MS was used to elute and acquire data. Representative total ion chromatograms of the cells and cell culture supernatants showed good separation and strong sensitivity of the established method (Supplementary Figure S1). PCA, which is an unsupervised pattern recognition analytical method based on the LC-MS data, was then used to visualize the trends among the groups. In the positive and negative ion mode, the PCA score plots of the liver samples showed a clear separation, which implied the perturbation of liver metabolic profiles among the three groups (Figures 4A,B). The model R2X parameters, which represent the model’s ability to interpret variables, were determined to be at 0.545 and 0.649 in the positive and negative ion mode, respectively, which indicates that 54.5 and 64.9% of the variables are used in building the analysis model in the positive and negative mode, respectively.

To improve the classification, OPLS-DA was introduced. The OPLS-DA score plots have been determined to show that the three groups were clearly separated, which suggested that significant metabolic changes were induced by an ethanol diet and were reversed by *L. reuteri* treatment (Figures 4C,D). This finding is consistent with the PCA result. To ensure the reliability of the results, permutation test is widely used in the OPLS-DA model of metabolomics analysis. The intercept of the R2 and Q2 parameters reached 0.699 and −0.767 in the positive ion mode, respectively, and 0.821 and −0.553 in the negative ion mode, respectively, which further indicated that overfitting did not occur (Figures 4E,F). The VIP value, which was obtained from the OPLS-DA model, was then used to identify metabolites using Student’s *t*-test to ensure that the metabolites selected were statistically significant. The compounds for which the VIP value was >1 and *p* < 0.05 were screened. In the positive and negative ion modes, 15 and 8 potential biomarkers were screened, respectively.

Twenty-three potential biomarkers were then identified in the search for MS/MS fragmentation patterns in HMDB and consisted of the following: 7 fatty acids and their metabolites and 11 lysophosphatidylethanolamine (LysoPE). In addition, five candidate biomarkers were observed that can be identified in the KEGG database with a fold change >1.5 or < 0.5; these included xanthosine, palmitic acid (PA, 16:0), and n-3 polyunsaturated fatty acids, such as linoleic acid (LA, 18:2), arachidonic acid (AA, 20:4), and docosapentaenoic acid (DPA, 22:5) (Table 1). Interestingly, all candidate biomarkers were found in the negative ion mode.

**TABLE 1:** The result of candidate biomarkers.

**Metabolite**	**VIP**	***p*-value**	**Fold Change(P/E)**	**RT (min)**	**SM**
Linoleic acid (18:2)	6.328	0.009	1.68	13.26	–
Arachidonic acid (20:4)	4.590	0.015	2.20	15.72	–
Docosapentaenoic acid (DPA, 22:5)	3.434	0.038	2.28	22.06	–
Xanthosine	1.779	0.001	2.01	5.08	–
Palmitic acid (16:0)	2.962	0.028	1.72	23.35	–

*RT, retention time; VIP, variable importance in projection; SM, scan mode; +, metabolites identified in the positive (ESI +) electrospray ionization mode; −, metabolites identified in the negative (ESI-) electrospray ionization mode. P/E, the probiotics group compared with the ethanol group.*

**FIGURE 5:**
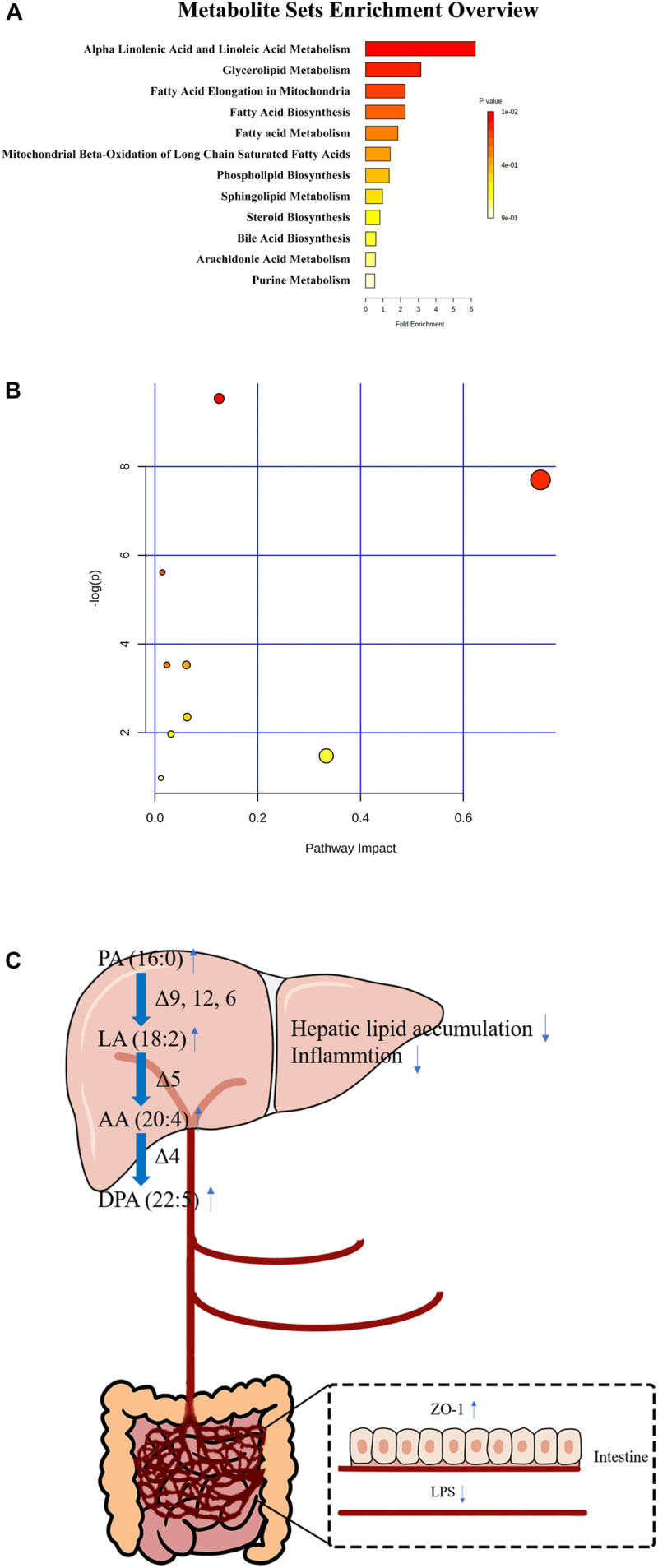
The enriched KEGG pathways **(A,B)** and the diagram of the protective effect of *L. reuteri* on ALD **(C)**.

To examine the effects of the candidate biomarkers on metabolic pathways, enrichment analysis of KEGG pathways was performed for the nine potential biomarkers that could be identified in the KEGG database. As shown in Figures 5A,B, alpha-linolenic acid and LA metabolism, glycerolipid metabolism, fatty acid elongation in mitochondria, fatty acid biosynthesis, fatty acid metabolism, mitochondrial beta-oxidation of long-chain saturated fatty acids, phospholipid biosynthesis, sphingolipid metabolism, steroid biosynthesis, bile acid biosynthesis, AA metabolism, and the purine metabolism pathways were found to be involved in the metabolic change between ethanol diet group and probiotics treatment group. The alpha-linolenic acid and linoleic acid metabolism pathways had the highest enrichment degree and involved linoleic acid (LA), arachidonic acid (AA), and 12(13)-EpOME. These findings suggested that fatty acid metabolism was disturbed by an ethanol diet and that this disturbance was reversed via *L. reuteri* treatment.

## Discussion

Alcoholic liver disease has been identified as the most common type of chronic liver disease and is the main cause of liver-related mortality worldwide, which results in a significant burden to society and a threat to human health (Rehm et al., 2013). Given that the currently used drugs provide a limited survival benefit, there is an urgent demand to discover a new potential therapy (Seitz et al., 2018). In recent years, the protective effects of probiotics on metabolic diseases have been widely examined. *L. reuteri* has been demonstrated to be beneficial in many digestive system diseases and metabolic diseases, such as colitis and type 2 diabetes (Gao et al., 2015; Dahiya et al., 2017). Thus, we performed a metabolomics analysis to examine the metabolic protective effects of *L. reuteri* on ALD.

To investigate whether *L. reuteri* exerts a protective effect on ALD, it was administered to C57BL/6 mice together with ethanol. H&E staining showed an amelioration of steatosis and inflammatory cell infiltration under *L. reuteri* treatment; meanwhile, oil red O staining showed less lipid droplet accumulation. Consistent with the histomorphology analysis, the levels of AST, ALT, TG, TCH, LPS, and TNF-α were reduced under *L. reuteri* treatment. Besides, ZO-1 and occludin IHC were performed and the results showed ZO-1 was suppressed by alcohol and reversed after treated by *L. reuteri* while occludin was not affected by ethanol diet. However, occludin was indicated decreased in the colon of ethanol fed mice (Mir et al., 2016). The mechanism needs to be explored in our next research. Studies of liver and intestinal inflammation suggested that *L. reuteri* treatment reduced the expression levels of hepatic IL-1β, IL-6, and TNF-α and promoted the expression of the anti-inflammatory cytokine IL-10 by suppressing the mitogen-activated protein kinase and NF-κB signaling pathways (Liu et al., 2012; Hsu et al., 2017). Hypercholesterolemic rats treated with a probiotics mixture containing *L. reuteri* have exhibited decreased lipid accumulation and reduced expression levels of cholesterol synthesis-related proteins, such as sterol regulatory element-binding protein 1 (SREBP1), fatty acid synthase, and acetyl-CoA carboxylase in the liver (Kim et al., 2017). These findings might explain the mechanism by which *L. reuteri* reverses liver injury and lipid accumulation.

To further examine the metabolic changes, metabolomics was introduced to comprehensively detect changes in the liver samples. PCA and OPLS-DA scores showed a significant difference in the metabolic characteristics among the control group, model group, and treatment group. Twenty-three potential biomarkers were then screened, including 7 fatty acids and their metabolites as well as 11 LysoPE; xanthosine, PA, LA, AA, and DPA were also identified as 5 candidate biomarkers with fold changes >1.5, which indicates a significant change in fatty acid metabolism. Their biological functions and related metabolic pathways can explain the protective effects of *L. reuteri*. Nine biomarkers that could be found in the KEGG database were detected by KEGG enrichment analysis in order to identify the possible pathways involved. The enrichment of PA and three polyunsaturated fatty acids (PUFA), LA, AA, and DPA was worthy of further attention.

Palmitic acid is a type of saturated long-chain fatty acids (LCFA). Chronic alcohol abuse reduces the production of LCFA via suppressing the bacterial genes involved in the biosynthesis of saturated fatty acids, including malonyl CoA:ACP acyltransferase (FabD; [EC:2.3.1.39]), 3-oxoacyl-[acyl-carrier-protein] synthase II (FabF; [EC:2.3.1.179]) and 3-oxoacyl-[acyl-carrier protein] reductase (FabG; [EC:1.1.1.100]), leading to the limit the proliferation of lactobacilli which metabolizes saturated LCFA (Chen et al., 2015). Meanwhile, alcohol-induced liver injury and steatosis were reversed after the supplementation of saturated LCFA via preventing gut leakiness and restoring the eubiosis.

Linoleic acid is an n-6 PUFA that can be metabolized to AA. Several meta-analyses and prospective cohort studies have shown that a higher level of LA was associated with a decreased risk of coronary heart disease, while another study on hyperlipidemia showed a hypolipidemic effect on mice fed a high-fat diet, which suggests its potential use for ameliorating fatty acid metabolism (Jang et al., 2008; Farvid et al., 2014; Marklund et al., 2019). Jina et al. examined associations among LA, AA, and the lipoprotein subclasses VLDL, LDL, and HDL and showed that high levels of LA and AA were significantly associated with a low level of large VLDL particles and a high level of large HDL particles (Choo et al., 2010). These findings might explain why the increased levels of LA and AA exerted protective effects on liver steatosis under *L. reuteri* treatment.

Chronic alcohol consumption can lead to a decrease in Δ-6, Δ-5, and Δ-9 desaturase activity, which is essential for the formation of AA (Risti-Medi et al., 2013). Despite the traditional view that AA is a substrate for pro-inflammatory eicosanoids, substantial evidence supports the concept that AA also serves as a precursor for a group of potent anti-inflammatory mediators. In addition to its ability to be transformed into the pro-inflammatory prostaglandin E2 and leukotrienes by cyclooxygenase (COX) and 5-lipoxygenase (LO), AA can also be transformed into lipoxins, resolvins, protectins, maresins, PGE1, and PGI2, which are found to represent a series of potent bioactive compounds that have been reported to have anti-inflammatory effects in chronic liver diseases, such as ALD, NAFLD, and cirrhosis (Das, 2019). Lactobacillus rhamnosus GG has been reported beneficial to ameliorating the reduction of AA induced by ethanol diet (Shi et al., 2015).

Docosapentaenoic acid is an n-3 PUFA that has been demonstrated to inhibit the expression of pro-inflammatory cytokines, such as TNF-α, IL-1β, and IL-6, and to improve the expression of the anti-inflammatory cytokine IL-10 (Tian et al., 2017; Zheng et al., 2019). Kimberly et al. found that the n-3 DPA-derived protectin (PDn-3 DPA) biosynthetic pathway regulated the differentiation of human monocytes, which altered the macrophage phenotype, efferocytosis, and bacterial phagocytosis (Pistorius et al., 2018). Remarkably, Zheng et al. (2019) demonstrated that DPA could lead to a decrease in the substrate needed for the synthesis of pro-inflammatory eicosanoids (PGE2 and LTB4) in a dextran sulfate sodium (DSS)-induced colitis model. This finding might explain why an increased level of AA did not lead to an increased level of pro-inflammatory eicosanoids in our study.

Increased levels of PUFAs prevent liver inflammation and lipid accumulation, which indicates that fatty acid metabolism is a therapeutic target for the management of ALD. An eradication of bacteria group was established via antibiotics (ampicillin, vancomycin, neomycin, and metronidazole) in our initially. However, the morality of mice is uncontrollable when gavaged with ethanol and we failed to collect the samples of this group. The effect of eradicating gut bacteria on ALD needs to be further explored. Besides, further studies on fatty acid metabolism in ALD are therefore needed.

In conclusion, all these findings suggested that *L. reuteri* treatment reversed the phenotype of ethanol-induced hepatitis and disorders of fatty acid metabolism (Figure 5C). We speculate that *L. reuteri* might exert a protective effect on ALD by ameliorating fatty acid metabolism. These findings provide evidence that *L. reuteri* may serve as a new therapeutic strategy for ALD.

## Data Availability Statement

The raw data supporting the conclusions of this article will be made available by the authors, without undue reservation.

## Ethics Statement

The animal study was reviewed and approved by Animal Care and Use Committee of Southwest Medical University.

## Author Contributions

S-P and T-Z designed and established the mouse model. T-Z finished manuscript-writing and performed data analysis. PT and Y-D performed histomorphological analysis. B-Q and HC collected tissue samples. W-F and M-H supervised the study and revised the manuscript. All authors contributed to the article and approved the submitted version.

## Conflict of Interest

The authors declare that the research was conducted in the absence of any commercial or financial relationships that could be construed as a potential conflict of interest.

## Supplementary Material

Supplementary Figure 1 Representative total ion chromatograms of the cells and cell culture supernatants.
