# Supplementary material for: Liver Metabolomics Reveals the Effect of Lactobacillus reuteri on Alcoholic Liver Disease
Source: Front Physiol. 2020 Nov 12;11:595382. doi: 10.3389/fphys.2020.595382 (PMC7689281; doi:10.3389/fphys.2020.595382)

Table 1 Weight of control and probiotics group

	Control group	Probiotics group
Body weight	22.16 ± 0.62	22.09 ± 0.81
Liver weight	1.160 ± 0.286	1.162 ± 0.159
Liver/body weight ratio	0.0524 ± 0.0006	0.0526 ± 0.0020

Table 2 Biochemical biomarkers of control and probiotics group

	Control group	Probiotics group
ALT	40.15 ± 3.67	39.62 ± 2.89
AST	88.33 ± 3.77	89.60 ± 6.11
TG	0.51 ± 0.12	0.48 ± 0.02
TCH	1.93 ± 0.13	1.86 ± 0.05

HE staining

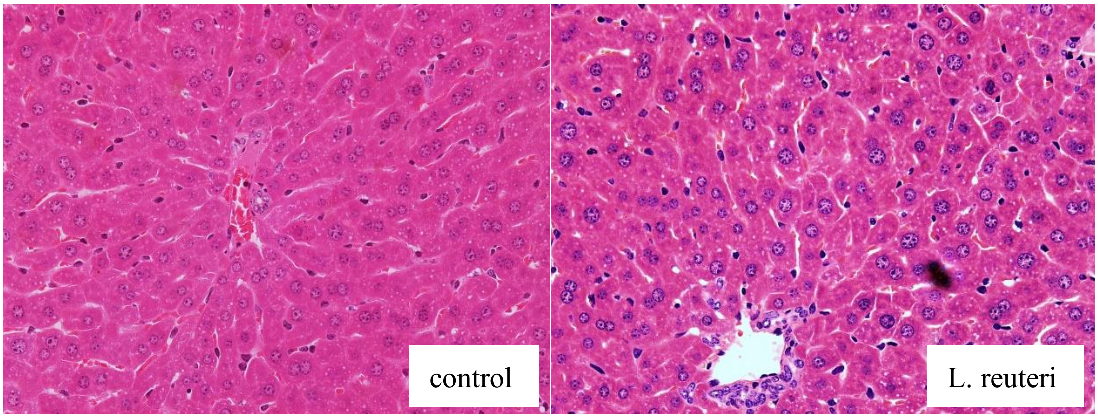

Oil red O staining

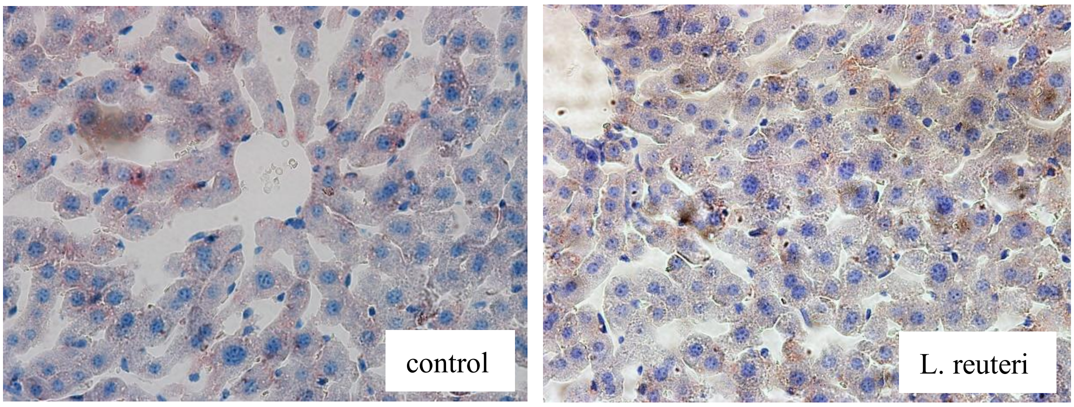

Supplement: Supplementary file 2 [file Image_2.pdf]
